# Impact of environmental factors on the bionomics of *Anopheles* mosquito vectors of zoonotic malaria: A narrative review

**DOI:** 10.1016/j.onehlt.2025.101141

**Published:** 2025-07-14

**Authors:** Rezki Sabrina Masse, Indra Vythilingam, Kimberly Fornace, Hidayatulfathi Othman, Xiaoyue Liu, Abdul Jabir Jaafar, Mohd Khadri Shahar, Nurul Asmaa Abdul Rahman, Ainul Huda Khairul Azman, Nantha Kumar Jeyaprakasam

**Affiliations:** a Medical Entomology Unit, Infectious Disease Research Centre, Institute for Medical Research (IMR), National Institutes of Health (NIH), Shah Alam, Selangor, Malaysia; b Biomedical Science Programme, Center for Toxicology & Health Risk Studies (CORE), Faculty of Health Sciences, Universiti Kebangsaan Malaysia, Kuala Lumpur, Malaysia; c Department of Parasitology, Faculty of Medicine, University of Malaya, Kuala Lumpur, Malaysia; d Saw Swee Hock School of Public Health, National University of Singapore, Singapore.

**Keywords:** *Anopheles*, Environmental factors, Land use, Malaria vectors, Non-human primates, Southeast Asia, Zoonotic

## Abstract

The increasing burden of non-human primates (NHP) malaria, driven primarily by *Plasmodium knowlesi*, poses a growing public health threat in many countries across Southeast Asia. Compounding this challenge, the emergence of other NHP *Plasmodium* species infecting humans, including *P. cynomolgi, P. inui, and P. fieldi*, introduces additional complexity to malaria elimination efforts, particularly in countries like Malaysia, Thailand, and Vietnam. A complex interplay among human populations, vector dynamics, and environmental factors influences the transmission and prevalence of this disease. This narrative review delves into the current vectors of NHP malaria in Southeast Asia, highlighting the crucial role of environmental determinants in shaping the bionomics of *Anopheles* mosquito vectors. Key environmental factors, such as temperature fluctuations, relative humidity, elevation, precipitation patterns, seasonality, and land use, play a pivotal role in shaping vector abundance and survival, ultimately influencing the transmission intensity of zoonotic malaria. Adopting a One Health approach, which recognizes the interconnections between human, animal, and environmental health, is crucial for unravelling these complex dynamics. Advancing this integrated framework will require continued research and understanding of vector ecology across diverse environmental settings and geographical regions. This review provides comprehensive information on vector bionomics in relation to the changing environmental factors, besides highlighting the importance of a multidisciplinary strategy that integrates vector surveillance, sustainable land management, and targeted public health interventions to inform effective, evidence-based malaria control efforts in Southeast Asia.

## Graphical abstract

**Figure:**
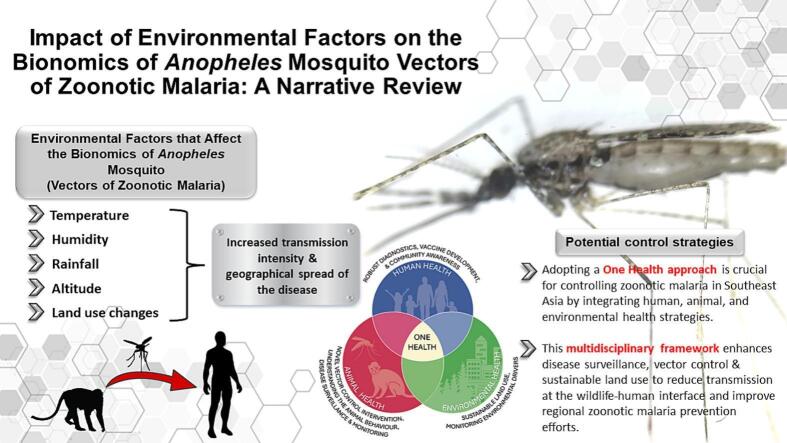

## 1 Introduction

Malaria continues to be a significant global health concern, despite the availability of comprehensive disease control strategies. In 2022, an estimated 249 million human malaria cases were reported across 85 malaria-endemic countries and regions worldwide, marking an increase of 5 million cases from the previous year [1]. Until recently, only five species of malaria parasites, namely *Plasmodium falciparum*, *Plasmodium vivax*, *Plasmodium malariae*, *Plasmodium ovale wallikeri*, and *Plasmodium ovale curtisi*, were known to infect humans. Although these parasites were discovered to have zoonotic origins from ape populations, they are now primarily transmitted among human populations [2]. However, *Plasmodium knowlesi*, a non-human primates (NHP) malaria of long-tailed macaques and pig-tailed macaques has been confirmed to infect humans, particularly in Southeast Asia countries where *P. falciparum* and *P. vivax* infections have significantly decreased [3] as well as in countries that have eliminated human malaria [1]. Since the first report of hundreds of human *P. knowlesi* cases in Sarawak, Malaysian Borneo, in 2004 [4], the incidence of *P. knowlesi* has markedly increased across Southeast Asia, except Timor-Leste, which has not reported cases thus far [5]. The World Health Organization (WHO) reported that Malaysia remained the primary source of *P. knowlesi* cases in Southeast Asia in 2022, with 90.5 % of cases, followed by Thailand and Indonesia, which contributed 3.1 % and 0.1 %, respectively [1]. Despite Malaysia reporting the highest absolute number of *P. knowlesi* cases in this region, Indonesia and Thailand experienced the most pronounced rate increase in that year [1]*.* Additionally, reports of natural human infections from other NHP malaria parasites, such as *P. cynomolgi* and *P. inui*, have added a new level of complexity to malaria elimination efforts in this region [5].

Numerous species of malaria parasites are found in NHP and can naturally infect humans with malaria [[6], [7], [8]]. These *Plasmodium* include *P. knowlesi, P. coatneyi, P. cynomolgi*, *P. inui*, *P. inui*-like, *P. simium*, *P. simiovale*, and *P. brasilianum* [9]. *Macaca fascicularis*, a long-tailed macaque, was identified as a natural reservoir of five zoonotic *Plasmodium* parasites, namely *P. knowlesi, P. fieldi, P. cynomolgi, P. coatneyi,* and *P. inui*, and has a wide geographical distribution among human populations in Southeast Asia regions [[10], [11], [12]]. Furthermore, various *Anopheles* species belonging to Leucosphyrus group were incriminated as vectors of *P. knowlesi* and other zoonotic malaria parasites in this same region [[12], [13], [14], [15], [16], [17], [18], [19], [20], [21], [22], [23]]. Notably, both the natural host macaque and the zoonotic vectors have a broad and overlapping geographical distribution among humans in several Southeast Asia countries.

Indeed, increased human knowlesi malaria cases are linked to deforestation and land cover changes, as the natural reservoirs and vectors were found to inhabit forests and forest fringes [24,25]. The expansion of these habitats, driven by both natural and anthropogenic factors, can disrupt the balance in which disease-carrying vectors and parasites thrive, increasing the risk of *P. knowlesi* transmission and other non-human primate *Plasmodium* species infecting humans [26,27]. Changes in the ecology, such as deforestation, lead to changes in the bionomic of the vector species in that area [28]. Each vector species occupies a specific ecological niche, and populations of vector species show distinct behavioural differences based on geographic region, suggesting adaptation to man-made environments [29]. Moreover, the human-biting behaviour of these vectors, which is influenced by factors such as human availability, flight range, biting frequency, and timing in relation to human habits, further shapes their role in disease transmission [30,31].

Numerous malaria vectors naturally exhibit significant irritability or repellency upon initial contact with residual insecticides, a behaviour that can influence their feeding patterns in response to vector control measures [32]. For example, a study in Malaysian Borneo found that the use of insecticide-treated nets (ITNs) led to a shift in *An. balabacensis* biting behaviour, with peak human biting time moving from 2100 to 2200 h to an earlier window of 1900–2000 h, along with an increased tendency for outdoor biting [33]*.* Substantial numbers of *An. balabacensis* have been documented biting outdoors during the early evening hours, a period when long-lasting insecticidal net (LLIN) use is typically low and human exposure risk is heightened, despite LLINs remaining Malaysia's principal malaria control strategy [20]. Although these changes in biting behaviour might be linked to insecticide pressure, further studies are needed to assess if they represent actual adaptations or result from other ecological or environmental factors. While vector behaviour has been extensively studied, the role of human behaviour in influencing mosquito exposure, particularly in the context of *P. knowlesi* infections, remains underexplored and warrants greater attention [34]. Human activities such as engaging in forest-related work, outdoor sleeping patterns, and land use practices play a significant role in influencing exposure risk, as they increase the likelihood of contact with both macaque hosts and mosquito vectors [35]. Concurrently, macaque activities, including their movement patterns, habitat preferences, and interactions with mosquito vectors, are critical ecological factors sustaining the zoonotic transmission cycle [36]. In parallel, variations in parasite genetic diversity across endemic regions are closely linked to transmission intensity and further shape the complexity of vector–host interactions [37].

Although there is increasing evidence linking the rise of NHP malaria in Southeast Asia to environmental changes, the complexities of how these factors influence vector bionomics remain inadequately addressed. Thus, this review aims to explore the relationship between environmental changes and the rise of zoonotic malaria in Southeast Asia, addressing the gaps in understanding how these factors influence vector bionomics. Key environmental variables, such as temperature, humidity, rainfall, altitude, seasonality, and land use, are analyzed for their impacts on vector proliferation, disease transmission intensity, and geographical distribution.

## 2 Vectors of NHP malaria in Southeast Asia countries

### 2.1 Distribution of the NHP malaria vectors in Southeast Asia

A comprehensive investigation of NHP malaria vectors was swiftly initiated following the report of widespread natural transmission of *P. knowlesi* in Kapit, Sarawak, East Malaysia, in 2004 [4]. Research on NHP vectors has predominantly been conducted in Malaysia, with additional studies in Indonesia [11], Thailand [12] and Vietnam [19]. Most *Anopheles* mosquito species from the Leucosphyrus group, along with some species from the Barbirostris and Umbrosus groups, have been identified as vectors of *P. knowlesi* and other NHP malaria parasites in Southeast Asian countries [[12], [13], [14], [15], [16], [17], [18], [19], [20], [21], [22], [23]]. These findings expand upon prior detailed reviews by Vythilinggam et al. [38]. To date, seven out of the 21 *Anopheles* species in the Leucosphyrus group have been incriminated as vectors for NHP malaria in Southeast Asia, with five of these species capable of transmitting all five zoonotic malaria species namely *P. coatneyi*, *P. cynomologi*, *P. fieldi*, *P. inui* and *P. knowlesi* (Table 1). The Leucosphyrus group is divided into three sub-groups: Hackeri, Leucosphyrus, and Riparis, with the Leucosphyrus sub-group further subdivided into the Dirus and Leucosphyrus complexes. This group is found throughout Southeast Asia, including the islands of Hainan and Taiwan, as well as southern India and Sri Lanka [39].

**Table 1:** Distribution of NHP malaria vectors in Southeast Asia Countries.

Group	Vector Species	NHP *Plasmodium* Species					Distribution	References
		*Pct*	*Pcy*	*Pfi*	*Pin*	*Pk*		
Leucosphyrus	*An. balabacensis*	/	/	/	/	/	Brunei, Indonesia, East Malaysia, Philippines	[20,21,[43], [44], [45], [46], [47]]
	*An. cracens*				/	/	Indonesia, West Malaysia, Thailand	[17,23]
	*An. dirus*	/	/		/	/	Cambodia, China, Laos, Thailand, Vietnam	[14,19,42]
	*An. hackeri*	/	/	/	/	/	Malaysia, Philippines, Thailand	[13]
	*An. introlatus*	/	/	/	/	/	East and West Malaysia, Indonesia, Thailand	[12,18,23]
	*An. latens*	/	/	/	/	/	Indonesia, East and West Malaysia, Thailand	[12,15,16,22,23]
	*An. sulawesi*				/		Indonesia	[11]
Barbirostris	*An. donaldi*					/	Indonesia, Laos, East Malaysia, Thailand	[21,45]
Umbrosus	*An. collesi*					/	East and West Malaysia, Brunei	[22]
	*An. roperi*	/	/			/	East and West Malaysia, Thailand, Cambodia, Nicobar Island of India	[22]

*Pct, P. coatneyi; Pcy, P.cynomolgi; Pfi, P. fieldi; Pin, P.inui; Pk, P. knowlesi*.

In Malaysia, the role of *An. latens* in the transmission of *P. knowlesi* malaria was first confirmed in Kapit, Sarawak [15,16]. Later studies incriminated *An. cracens* as the vector of knowlesi malaria in Kuala Lipis, Pahang [17], *An. introlatus* in Selangor [18] and *An. balabacensis* in Sabah [20]. Recently, *An. introlatus* has also been confirmed to harbor four additional zoonotic malaria species; *P. coatneyi*, *P. cynomolgi*, *P. fieldi*, and *P. inui*—in its salivary glands, found in Johor, Negeri Sembilan, Pahang, and Perak, Peninsular Malaysia [23]. *Anopheles dirus* was the predominant vector of human malaria and knowlesi malaria in Vietnam [40,41]. Meanwhile, in Thailand, experimental studies confirmed that laboratory-reared *An. dirus* mosquitoes can transmit *P. knowlesi* from human-infected blood, indicating the potential for human-mosquito-human transmission [42]. Besides, recent studies have incriminated *An. latens* and *An. introlatus* as natural vectors of NHP malaria in Southern Thailand [12]. Thus, further investigation of the heterogeneity in vector species within Thailand is essential. In Indonesia, only one vector study successfully detected the NHP malaria parasite, *P. inui*, in the thorax of *An. Sulawesi* [11]. However, further entomological studies on this member of the Leucosphyrus group are needed.

In Sarawak state, Malaysian Borneo, *Anopheles* species other than the Leucosphyrus group have been incriminated as vectors for knowlesi malaria, including *An. donaldi* from the Barbirostris group and *An. collesi* and *An. roperi* from the Umbrosus group. However, in all the newly discovered vector species from the non-Leucosphyrus group, parasites were detected through PCR analysis of the salivary glands and the whole mosquito. No microscopic observations were made to confirm the presence of sporozoites and oocysts [21,22]. Therefore, the incrimination of natural vectors from the non-Leucosphyrus group in transmitting NHP malaria in the Southeast Asia region remains inconclusive. Indeed, molecular detection methods have enabled the accurate identification of *P. knowlesi* and other NHP malaria parasites in wild mosquitoes, providing critical insights into vector ecology. This precise detection is essential for incriminating specific vector species and assessing their potential role in the zoonotic malaria transmission from non-human primates [38].

### 2.2 Larval biology of NHP malaria vectors

Understanding the distribution and larval habitats of mosquitoes is extremely important for controlling the spread of disease by vectors. Generally, the larval habitat requirements of NHP malaria vectors vary slightly among different mosquito species (Table 2). The *Anopheles* Leucosphyrus group mosquitoes, typically found in forested and agricultural settings [18], have their larval habitats in highly shaded, clean, natural water pockets or puddles near rivers [48]. The larval habitats of *An. latens* and *An. balabacensis* are mainly found in shaded temporary pools and natural containers, whether the water is clear or turbid, located on forest floors [48]. On the other hand, *An. sulawesi,* which is a potential carrier of the NHP malaria parasite, *P. inui,* in Indonesia was observed to exhibit varying breeding preferences depending on the location, favouring shaded breeding sites near human residences and non-shaded sites farther from human settlements [11,49]. Generally, all species within the Leucosphyrus group displayed similar breeding preferences, except for *An. hackeri* [38]*.* In inland forest areas, *An. hackeri* was observed breeding in split bamboo, while in coastal regions, it preferred the leaf base cavities of the nipah palm for breeding [50]. Research on the larval breeding habitats of zoonotic malaria vectors in Southeast Asia is notably scarce, especially considering the ongoing climate and environmental changes. Identifying and understanding these habitats is critical in developing effective vector control strategies. Targeting these habitats can help reduce mosquito populations and minimize malaria transmission risks substantially.

**Table 2:** Larval habitat characteristics of NHP malaria vectors in Southeast Asia.

Vector Species	Light intensity	Turbidity	Water movement	Larval habitat	References
*An. balabacensis*	Typical heliophobic	Clear, turbid, fresh water, muddy pool	Still or flowing	Streams, pools, puddles, wheel ruts, hoof prints	[51,52]
*An. cracens*	Heliophobic	Fresh water	Still or stagnant	Pools, puddles, Elephant and other animal footprints	[29,51]
*An. dirus*	Heliophobic	Clear, turbid, fresh water	Still or stagnant	Small streams, pools, wells, dips in the ground, borrow pits, wheel ruts, hoof prints	[29,51]
*An. donaldi*	Typical heliophobic	Clean, fresh water	Still or stagnant	Small streams, ground pools, occasionally rice fields, and open marshlands	[53,54]
*An. hackeri*	Heliophobic	Clean non-saline water, but found in water cointaning up to 4 % sea-water	Still or stagnant	Elephant footprints, In split bamboo and cavities at the leaf base of nipah palm	[29,50]
*An. latens*	Heliophobic	Clear, turbid, fresh water, muddy pool	Still or stagnant	Small streams, seepage streams, pools, wheel ruts, hoof prints	[29,51,55,56]
*An. sulawesi*	Both heliophobic and heliophilic	Fresh water	Still or flowing	River flow, puddle river	[11,49]

### 2.3 Biological characteristics of adult NHP malaria vectors

Slight differences in the biological characteristics of adult NHP malaria vectors play a critical role in shaping their behaviour and capacity for disease transmission (Table 3). Among these vectors, *An. hackeri* is primarily known to feed on monkeys and rarely bite humans [14]. While *An. latens* is acknowledged as a vector of human malaria in East Malaysia [55]. Studies indicate that this species shows a greater preference for monkeys over humans [17]. Currently, *An. latens* has been identified as a vector for multiple NHP malaria parasites in both East and West Malaysia [16,17,24]. In contrast, *An. cracens*, the primary vector of knowlesi malaria in West Malaysia, demonstrates a preference for both monkeys and humans [18]. Interestingly, unlike *An. latens*, *An. cracens* has only been found to harbor NHP malaria parasites, particularly *P. knowlesi* and *P. inui*, but not with human malaria parasites [18,24]. Recently, *An. sulawesi* has been identified as a potential vector of zoonotic malaria in Indonesia, following the discovery of *P. inui* in its thorax. However, further confirmation is required to investigate the role of *An. sulawesi* as the main vector of zoonotic malaria in this region [12]. With regards to the non-Leucosphyrus group, further investigation into their feeding preferences is crucial for determining host specificity, while blood meal analysis can further elucidate their feeding behaviour and transmission potential. These discoveries underscore the complexity of zoonotic malaria transmission and emphasize the need for continued research to clarify the roles of various mosquito species across different geographical regions in sustaining and spreading the disease.

**Table 3:** Information on the biological variations among adults of zoonotic malaria vectors in Southeast Asia**.**

Vector Species	Country	Habitat: Peak biting time [Indoor (I),Outdoor (O)]	Sporozoite rate (%)	Entomological inoculation rate (EIR) (%)	Life Expectancy	Vectorial Capacity	Host Preference	References
*An. latens*	Narathiwat Province, Southern Thailand	Forest fringe: 1800-1900 h [O]	NA	NA	NA	NA	HBR [1.625]	[12]
	Kapit District, Sarawak, East Malaysia	Forest: 1900 -2000 h; Farm: 0100-0200 h [O]	Forest: 1.4 Farm: 0.7	Forest: 14.1 Farm: 11.98	Forest: 4.7; Farm: 7.2; Longhouse: 7.2	Forest: 0.60;Farm: 2.86;Longhouse: 0.85	MBT: HBT 1.0:1.3 (Simio-anthropophilic)	[16]
	Kelantan and Johor state, West Malaysia	Forest and Village: 1900-2000 h [O]	Village: 10.00(0.52–45.88)	Village: 0.06	Forest: 7.4; Village: 8.4	Forest: 0.34; Village: 0.53	NA	[23]
*An. introlatus*	Narathiwat Province, Southern Thailand	Forest fringe: 2000–2100 h [O]	NA	NA	NA	NA	Simio-anthropophilic	[12]
	Johor, Pahang, Kelantan and Perak states, West Malaysia	Forest, Forest fringe and Village: 2000-2100 h [O]	Forest: 4.23 (1.98–8.47); 12.50 (0.66–53.32); 10.00 (0.52–45.88); Forest fringe: 14.29 (0.75–57.99)	Forest: 0.27; 0.04; 0.06; Forest fringe: 0.06	Forest: 7.9; 8.4; 8.9; Forest fringe: 10.4	Forest: 4.6; 0.44; 0.42; Forest fringe: 0.53	NA	[23]
*An. balabacensis*	Ranau and Keningau District, Sabah, East Malaysia	Forest edge and Plantation: 1900–2000 h [O]	NA	NA	NA	NA	NA	[45]
	Kinabatangan District, Sabah, East Malaysia	Forest fringe: 1900-2000 h [O] (76 %); 2200-2300 h [I] (24 %)	NA	NA	NA	NA	NA	[34]
	Banggi Island, Sabah, East Malaysia	Farm and Secondary forest: 1800-2000 h [O]	Farm: 1.93 (0.85–4.12); Secondary forest: 3.42 (1.91–5.93)	Farm: 0.24; Secondary forest: 0.13	Farm: 6.7; Secondary forest: 7.0;	Farm: 3.36; Secondary forest: 3.85	NA	[20]
	Kudat District, Sabah, East Malaysia	Village: 1800-2000 h [O]	1.03 (0.45–2.20)	0.09	5.4	2.5	NA	[20]
	Palawan Island, Philippines	Forest: 2000-0300 h [O]	12.5	NA	NA	NA	NA	[14]
	Lombok Island, Indonesia	Forest: 1900-2100 h [O]	NA	NA	NA	NA	NA	[57]
*An. dirus*	Ninh Thuan Province, South- Central Vietnam	Forest and Forest fringe: 2000-2200 h; 0000-0200 h [O]	1.67	NA	NA	NA	NA	[19]
*An. cracens*	Kuala Lipis, Pahang State, West Malaysia	Forest fringe: 2000-2100 h [O]	Fruit orchard: 0.6 Forest: 2.9	0.08	8.6	1.09	Simio-anthropophilic	[17]
	Pahang State, West Malaysia	Forest: 1900-2000 h [O]	5.41 (0.94–19.53)	0.12	7.4	1.46	NA	[23]
*An. hackeri*	Selangor State, West Malaysia	Coastal area: Not known since most are attracted to monkeys rather than humans	0.14	NA	NA	NA	Zoophilic	[50]
	Selangor State, West Malaysia	Coastal area: Not known since most are attracted to monkeys rather than humans	0.7	NA	NA	NA	Most attracted to monkeys at canopy level in the mangrove forest. Not attracted to humans.	[13]
*An. sulawesi*	Buton Island, Southeast Sulawesi, Indonesia	Forest fringe: 1900-0700 h [O]	12.5	0.98	NA	NA	NA	[11]
*An. donaldi*	Ranau and Keningau District, Sabah, Malaysian Borneo	Forest edge and Plantation: 1800-1900 h [O]	NA	NA	NA	NA	NA	[45]
	Kinabatangan District, Sabah, East Malaysia	Forest fringe: 1800-2100 h [O] (90 %); 1800-2100 h [I] (10 %)	NA	NA	NA	NA	NA	[34]
*An. collesi*	Betong District, Sarawak, Malaysian Borneo	Secondary forest: 1700–1800 h; 0800–0900 h [O]	NA	NA	NA	NA	NA	[22]
*An. roperi*	Betong District, Sarawak, Malaysian Borneo	Secondary forest: 1800–1900 h; 0700-0800 h [O]	NA	NA	NA	NA	NA	[22]

HBR: Human biting rate, MBT: Monkey baited trap, HBT; Human baited trap, HLC: Human Landing catches, CBT: Cattle baited trap.

## 3 Effect of environmental factors on the bionomics of *Anopheles* mosquito vectors of NHP malaria

**Fig. 1:**
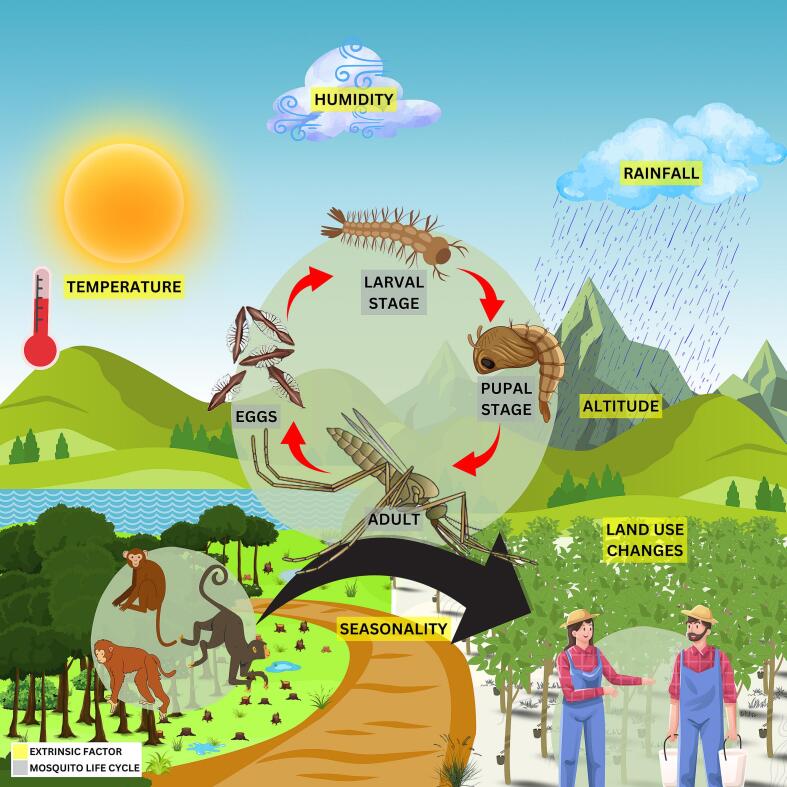
Interaction of extrinsic factors with the life cycle of NHP malaria vectors**,** highlighting their influence on vector-host interactions between macaques and humans.

**Fig. 2:**
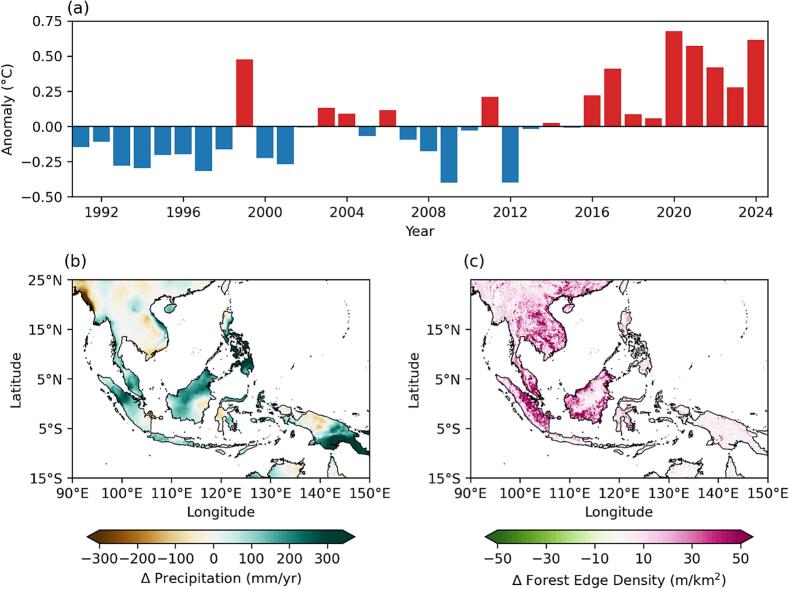
Climate and environmental changes in Southeast Asia (15°S – 25°N, 90°E – 150°E) in recent decades. (a) Near-surface temperature anomaly (°C) from 1991 to 2024, based on the 1991–2020 climatology [64]; (b) Changes in annual precipitation (mm/yr) between the 1991–2020 and 1960–1990 climatologies [64]; (c) Changes in forest edge density (m/km^2^) between 2000 and 2023 [65].

Multiple environmental factors influence the life cycle and distribution of *Anopheles* mosquito, thereby influencing vector–host interactions between humans and wildlife (Fig. 1). These factors, including temperature, humidity, rainfall, altitude, seasonality, and land use changes that significantly shape the dynamics of *Anopheles* populations [25,28,[58], [59], [60], [61]]. This complex interplay underscores the One Health concept and highlights the importance of integrated approaches that consider environmental, animal, and human health in efforts to understand and control zoonotic malaria transmission. In recent decades, climate change and anthropogenic activities have led to substantial environmental shifts across Southeast Asia (Fig. 2), including unprecedented heatwaves, with Laos recording its highest-ever temperature of 43.5 °C in Luang Prabang in May 2023 [62]; shifts in rainfall patterns with more frequent extreme precipitation events projected [63]; and extensive deforestation largely driven by palm oil expansion, continues at an alarming rate. These environmental changes will have profound implications for mosquito habitats, vector distribution, and malaria transmission dynamics. Understanding these factors is essential for predicting zoonotic malaria transmission patterns and informing effective vector control strategies.

### 3.1 Temperature

Temperature is a key determinant in the biology of both immature and adult female mosquitoes, profoundly affecting their development, survival, and capacity to transmit disease [66]. A study in Thailand found that laboratory-reared *An. dirus* larvae grew significantly faster at 30 °C, completing their development in about 7 to 8 days, compared to a longer duration at 23 °C. However, at the higher temperature (30 °C), adult *An. dirus* mosquitoes exhibited reduced fecundity and were smaller in size [67]. Mosquito size, which is affected by temperature, plays a critical role in determining several key epidemiological factors, such as longevity, gonotrophic cycle length, biting rate, immunocompetence, and infection intensity [68]. These traits, in turn, influence both parasite development [69] and vector survival [70]. As such, temperature also has a direct impact on the extrinsic incubation period (EIP), the time it takes for the malaria parasite to develop within the mosquito. Higher temperatures shorten the EIP, allowing the parasite to reach an infectious stage more quickly, thereby increasing the likelihood of malaria transmission [71]. Furthermore, an increase in temperature accelerates blood meal digestion and shortens the gonotrophic cycle, thereby enhancing the mosquito's vectorial capacity [72]. This dual impact on their development and functional efficiency directly affects their malaria transmission potential and the length of the transmission period. A similar trend was observed in the distribution of *An. balabacensis* collected in the Kudat district of Sabah, Malaysia, which exhibited a significant negative correlation with temperature [44]. However, extreme temperatures can adversely affect mosquito survival by either accelerating metabolic rates or causing mortality, indicating that both high and low temperature extremes can limit the mosquito population. As highlighted by Mordecai et al. [73], malaria transmission is confined to temperatures between 17 °C and 34 °C, driven by the physiological constraints of both mosquitoes and parasites. Thus, while moderate warming can facilitate mosquito-borne transmission, extreme temperatures may reduce their efficacy as vectors. Overall, temperature significantly impacts the distribution, development, and transmission potential of NHP malaria vectors. While temperatures are rising (Fig. 2a), spatial heterogeneity in both baseline temperature and warming trends complicates predictions of mosquito responses. Indeed, local factors such as land cover, vegetation types, and topography can create diverse thermal conditions, leading to region-specific vector dynamics [74]. Additionally, findings from controlled laboratory studies may not fully translate to natural settings, where mosquitoes interact with complex ecological and environmental factors. Thus, the net impact of global warming on malaria transmission remains context-dependent and requires further investigation.

### 3.2 Humidity

The humidity level notably affects mosquito vectors' distribution, particularly the forest-dwelling *Anopheles* Leucosphyrus group, which transmits NHP malaria and thrives in shaded, humid, and moist environments [75]. High humidity levels are essential for the survival and reproduction of mosquitoes, as it prolongs their lifespan and enhances their ability to find suitable breeding sites. *Anopheles* mosquitoes tend to thrive in environments with relative humidity above 60 %, supporting both larval development and adult survival [76]. Increased humidity also promotes the availability of stagnant water sources, which are crucial for mosquito breeding, especially in tropical and subtropical regions where *P. knowlesi* transmission occurs [77]. In contrast, low humidity can lead to desiccation, reducing mosquito survival and thus limiting the geographic range of *Anopheles* populations [78]. However, a study showed that relative humidity did not significantly impact *An. balabacensis* collections, likely due to microclimate effects and the use of humidity data from meteorological stations distant from the sampling site [44]. This highlights the importance of localized data collection to accurately assess environmental factors affecting vector populations and disease transmission dynamics. Humidity also affects the malaria parasite's development within the mosquito vectors. Mosquitoes are more active and take more blood meals in high-humidity environments, thereby increasing the chances of disease transmission to humans [58]. Conversely, dry conditions often restrict mosquito activity and reduce the probability of vector-human contact [77]. Therefore, regions with sustained high humidity, such as tropical rainforests in Southeast Asia, are more likely to experience high levels of *P. knowlesi* and other NHP malaria species transmission due to the favourable conditions for mosquito vectors. Indeed, humidity significantly influences both the distribution of NHP malaria vectors and the efficiency of disease transmission, making it a key factor in the ecology of malaria transmission.

### 3.3 Rainfall

Rainfall in Southeast Asia is a key driver of *Anopheles* mosquito populations and NHP malaria transmission, as it directly influences mosquito breeding habitats and vector dynamics. Understanding the climate modulators that shape rainfall at different temporal scales—ranging from intra-seasonal to annual variability—is crucial for predicting disease outbreaks and improving early warning systems [79]. At the interannual scale, the El Niño-Southern Oscillation (ENSO) plays a critical role in influencing malaria risk by altering rainfall patterns. El Niño years are typically characterized by drier conditions, while La Niña, in contrast, brings above-average rainfall [80]. Meanwhile, at the seasonal scale, the monsoon system governs rainfall distribution, with heavy precipitation during the wet monsoon creating stagnant water bodies that serve as ideal mosquito breeding sites [60]. This, in turn, drives a substantial increase in vector populations, and consequently raises the risk of mosquito-borne diseases, including zoonotic malaria transmission [77]. These water bodies are particularly crucial for the reproduction of mosquito species in forested, rural, and agricultural areas [18].

On sub-seasonal to intra-seasonal timescales, short-term rainfall fluctuations driven by the Madden-Julian Oscillation (MJO) can trigger active and break periods in monsoon rainfall [81]. These short-term variations influence mosquito population dynamics, as rainfall surges can accelerate breeding, while extended dry spells may limit larval development [60,77]. However, the impact of seasonality is not uniform across countries and varies according to local ecological and climatic conditions such as rainfall intensity [44,82]. These seasonal fluctuations complicate efforts to predict and control NHP malaria outbreaks in the region. In contrast, during the dry season, mosquito populations and transmission rates typically decline due to the reduced availability of breeding habitats and lower humidity levels, which limit mosquito survival [72]. Nevertheless, the stable temperature characteristic of tropical regions supports consistent mosquito activity throughout the year, enabling *P. knowlesi* transmission even outside the peak wet season [83]. This phenomenon underscores the complexity of malaria transmission dynamics in tropical climates, where seasonality alone does not fully account for transmission patterns. Instead, more nuanced models incorporating local ecological factors and year-round vector activity are necessary to improve the prediction and control of *P. knowlesi* malaria outbreaks.

In recent decades, while many regions in Southeast Asia have experienced increased rainfall (Fig. 2b), the nature of this increase varies. Some areas report more intense rainfall events, while others experience a higher frequency of drought-wet transitions. The strong link between rainfall and vector proliferation is further underscored by higher parity rates observed in *An. donaldi* and *An. balabacensis* during peak rainfall months [34]. In Sabah, this relationship is supported by evidence showing a correlation between increased rainfall and a subsequent rise in *P. knowlesi* malaria cases after a three-month delay [26]. However, excessive rain can disrupt this dynamic by washing away smaller breeding sites and altering environmental conditions, such as lowering temperatures, which can hinder mosquito survival and reduce disease transmission potential [84]. Studies have demonstrated that changes in rainfall intensity and seasonality can drive fluctuations in *P. knowlesi* case frequencies [61]. This intricate interplay between rainfall and vector ecology underscores its critical role in shaping zoonotic malaria outbreaks in Southeast Asia, but the specific effects of these varied rainfall patterns on *Anopheles* populations remain poorly understood and warrant further investigation. Furthermore, the well-established association between rainfall and the prevalence of *P. falciparum* and *P. vivax* malaria [[85], [86], [87]] has proven valuable for early warning systems and epidemic prediction [88,89]. Incorporating multi-scale rainfall influences—such as monsoon cycles, MJO activity, and ENSO forecasts—into zoonotic malaria surveillance can help public health programs better anticipate vector population surges and malaria transmission peaks, enabling more effective and proactive intervention strategies for *P. knowlesi* and other NHP malaria control.

### 3.4 Altitude

The distribution and ecology of *Anopheles* mosquitoes, particularly species that transmit *P. knowlesi*, are significantly influenced by elevation and temperature [58]. Altitude and temperature are generally inversely related; higher elevations typically have cooler climates, which create less favourable environmental conditions for the survival and reproduction of mosquitoes [90,91]. Spatial analysis studies conducted in Malaysia indicate that forested regions with high elevation and lower temperatures exhibit a marked decrease in vector abundance, thereby reducing the likelihood of zoonotic malaria transmission. In contrast, the predictive mapping reveals that areas with low elevation and moderate temperatures are more likely to support higher vector distributions [58]. These findings underscore the critical role of environmental factors in shaping the ecology of zoonotic malaria vectors and provide essential guidance for developing region-specific control and prevention strategies across Southeast Asia. While altitude currently serves as a natural barrier to transmission, climate change is driving shifts in mosquito distribution. Warming temperatures enable vectors to inhabit higher altitudes, potentially expanding the range of *P. knowlesi* and other zoonotic malaria species transmission into previously unaffected regions [92]. This altitudinal shift in vector distribution could increase the burden of zoonotic malaria in previously considered low-risk areas.

### 3.5 Land use changes

Changes in land use, especially deforestation driven by agricultural expansion and urbanization, have profoundly influenced the distribution of *P. knowlesi* vectors and increased the risk of disease transmission in Southeast Asia [25,28]. The large-scale removal of forest cover disrupts the ecological balance, particularly affecting forest-dwelling *Anopheles* mosquitoes of the Leucosphyrus group, which are the primary vectors of *P. knowlesi* [45]. Spatiotemporal analyses highlight that among the Southeast Asia countries, Malaysia and Indonesia are experiencing the highest rates of forest loss, primarily due to the rapid expansion of oil palm and rubber plantations [93]. As indicated in Fig. 2c, extensive forest clearing has directly led to increased forest edge densities across Southeast Asia over the past two decades, particularly in Thailand, Malaysia, and Indonesia. This landscape fragmentation enhances vector habitat suitability and increases human exposure to both *Anopheles* mosquitoes and macaques [93]. A study utilizing GPS tracking of local human movement patterns found that over 90 % of *An. balabacensis* infectious bites were predicted to occur in peri-domestic areas near forest edges rather than deep forest interiors, highlighting a significantly elevated risk of *P. knowlesi* exposure along these translational zones [94]. Additionally, areas near both secondary forests and human settlements exhibited the highest probabilities of human exposure, emphasizing the critical role of ecotones in facilitating disease transmission [94]. Supporting this, Brant et al. [95] suggested that changes in *P. knowlesi* vector abundance and distribution could be attributed to the increased availability of larval habitats resulting from land-use changes. This hypothesis is further reinforced by recent studies conducted in Malaysian Borneo, which have demonstrated that anthropogenic land-use alterations create more suitable habitats for *An. balabacensis* larvae, thereby potentially contributing to the higher incidence of *P. knowlesi* in humans [27]. In line with these findings, Chua et al. [44] reported that *An. balabacensis* shows strong adaptability to anthropogenic environments, as reflected in its high densities and observed breeding sites in oil palm plantations near human habitations. Most recently, *An. balabacensis* was found to be abundant and predominantly harboured two species of NHP malaria parasites, *P. inui* and *P. fieldi*, in degraded forest areas, in contrast to its lower presence in native forests, oil palm estates, and eucalyptus plantations [96]. Beyond altering mosquito habitats, deforestation and land-use change also modify the local microclimate, influencing key environmental factors such as temperature, humidity, and rainfall patterns, all of which affect *Anopheles* vector behaviour and survival in an indirect way [97]. Forest clearance and agricultural expansion tend to create warmer, drier environments, reducing overall humidity levels while increasing temperature extremes. These shifts can accelerate mosquito development rates, shorten gonotrophic cycles, and increase biting frequency. Additionally, deforestation disrupts regional rainfall patterns, potentially leading to localized droughts or more unpredictable precipitation, which can alter the availability of mosquito breeding sites [97,98]. Together, the direct and indirect impacts of land-use change can contribute to the expansion of *Anopheles* mosquito breeding, while also altering temperature and humidity conditions that enhance larval survival and development.

## 4 Implications of environmental factors on malaria transmission

### 4.1 Increased transmission intensity

Environmental changes, whether natural or anthropogenic, can significantly impact the transmission intensity of knowlesi malaria across Southeast Asia by influencing vector ecology, host availability, and human-vector interactions [26,60]. Among these factors, human-driven land-use changes have been strongly linked to the rising incidence of *P. knowlesi* infections, as they modify the distribution and abundance of its vectors by creating more suitable larval habitats [27]. Notably, *An. balabacensis*, a highly efficient vector, has demonstrated increased densities in oil palm plantations near human settlements [44] and degraded forest habitats [96]. These altered landscapes are simultaneously occupied by *P. knowlesi* reservoir macaques [47], establishing an ecological interface that facilitates zoonotic spillover. The close coexistence of vectors and reservoir hosts in these modified environments intensifies the transmission dynamics of the disease. Furthermore, environmental modifications such as deforestation and land-use changes can fragment habitats, increasing human exposure to *P. knowlesi* by enhancing the overlap between human populations, mosquito vectors, and macaque hosts. For instance, a study in Sabah, Malaysia, demonstrated that habitat fragmentation and proximity to agricultural areas significantly affect the distribution and density of *An. balabacensis* larvae, creating conditions that favour higher vector populations and elevate the risk of transmission [27]. High-resolution remote sensing data have shown that fine-scale land-use changes can generate optimal breeding habitats for mosquitoes, further intensifying transmission in fragmented and agricultural landscapes [27].

In addition, warmer temperatures further exacerbate this effect by accelerating the parasite's extrinsic incubation period [71], thereby enhancing transmission efficiency and sustaining endemicity in affected regions. Environmental stratification analyses have also indicated that certain land cover types and configurations are associated with elevated *P. knowlesi* exposure risk [94]. Moreover, vectors such as *An. dirus* have adapted to man-made environments like orchards and plantations, underscoring how human activity creates conditions that can increase vector densities and malaria transmission potential [99]. Collectively, these findings underscore the critical role of environmental factors in intensifying malaria transmission and highlight the urgent need for integrated land management strategies that consider the ecological impacts of human activities to effectively mitigate the risks associated with zoonotic malaria.

## 5 Future direction

Future research on NHP malaria should prioritize longitudinal entomological studies in more Southeast Asian countries affected by the disease, as most existing studies are largely focused on Malaysia and a few other neighbouring nations. Expanding research efforts regionally will help assess changes in vector abundances over time, particularly in relation to seasonal variations, ultimately improving our understanding of regional differences and enhancing control strategies. A key aspect of this research is determining whether peak vector abundance consistently occurs between March and April or if shifting environmental conditions, such as climate change, have altered this temporal pattern. To achieve this, a comprehensive analysis of historical data, combined with ongoing monitoring, is essential for identifying long-term trends in vector populations and detecting emerging shifts in their seasonal dynamics. This deeper understanding is crucial for refining vector control strategies, predicting changes in NHP malaria transmission patterns, and informing public health policies.

**Fig. 3:**
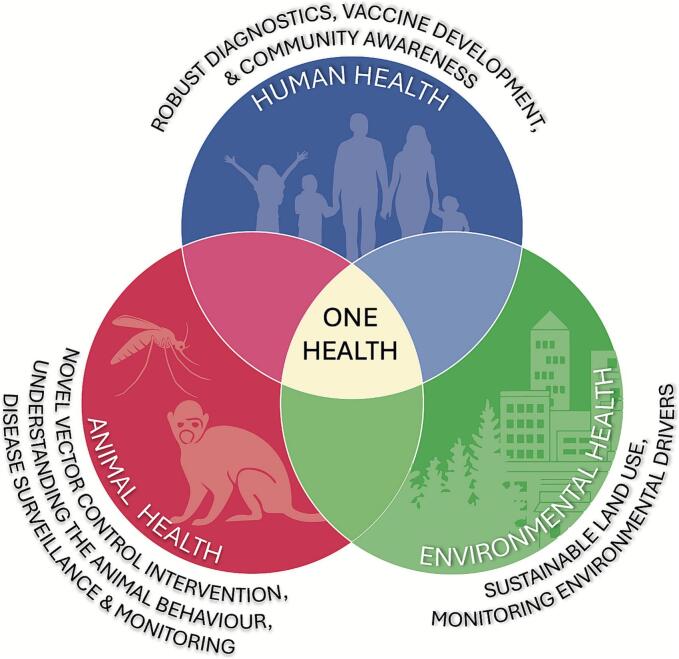
One Health Approach in Controlling NHP Malaria Transmission.

In this context, adopting a One Health approach, which integrates human, animal, and environmental health perspectives, is essential for developing sustainable and effective strategies to control zoonotic malaria (Fig. 3). Given the critical roles of *Anopheles* mosquitoes and macaques in sustaining zoonotic malaria transmission cycles, a multidisciplinary approach is required to address the complex interactions among humans, animals, and the environment. Implementation of novel vector control strategies, enhanced surveillance and monitoring of non-human primate reservoirs and mosquito vectors, and a deeper understanding of animal behaviour are vital to mitigating transmission risks. Simultaneously, promoting environmental health through sustainable land-use practices and continuous monitoring of ecological drivers can reduce vector breeding habitats and disrupt transmission dynamics. Equally important are advances in human health, including the development of reliable diagnostic tools, effective vaccines, and comprehensive community awareness initiatives, all of which contribute to improved prevention, early detection, and timely control efforts. Collectively, these efforts form the foundation of an integrated One Health framework, offering a holistic and evidence-based strategy to better understand the ecology of zoonotic malaria, enhance predictive capacity, and inform the implementation of context-specific interventions that address both human and sylvatic transmission pathways.

Moving forward, operationalizing the One Health approach is critical for effectively addressing the growing threat of zoonotic malaria in Southeast Asia. Future strategies should prioritize the establishment of integrated surveillance systems that concurrently monitor human malaria cases, macaque reservoir populations, and *Anopheles* vector dynamics to facilitate early detection and rapid response. Research efforts must focus on the development of innovative, ecologically tailored vector control interventions that account for the behavioural and biological diversity of local vector species, supported by land management practices that limit habitat disruption and control vector proliferation. Additionally, insights into macaque movement and resting behaviours in malaria-prone areas, along with an understanding of the spatial and temporal convergence of mosquito, human, and macaque activities, are essential for identifying high-risk transmission zones. Building on this, human behavioural factors, including occupational and recreational exposure, cultural norms, mobility patterns, and perceptions of risk, must also be systematically integrated into the planning and design of interventions. This integration requires participatory engagement with affected communities to ensure that proposed strategies are not only evidence based but also culturally appropriate, feasible, and sustainable over time. National governments play a central role in leading these initiatives through cross-sectoral coordination and adequate resource allocation. At the same time, regional and multi-country collaboration is essential to support harmonized policies, facilitate transboundary vector control efforts, and encourage the exchange of surveillance data, technical expertise, and best practices. By embracing these interdisciplinary and multilevel strategies, the One Health framework can be effectively translated into actionable policies and public health programs. This integrated approach holds significant promise for reducing zoonotic malaria transmission and accelerating progress toward malaria elimination across the Southeast Asian region. Indeed, for the One Health system to work, there needs to be great political will.

Future studies should explore the interactions between environmental factors such as climate variability, habitat modifications, land use changes, and the genetic adaptations of *Anopheles* mosquitoes involved in NHP malaria transmission. These investigations should also examine how changing environmental conditions drive evolutionary adaptations in mosquito populations and their implications for transmission dynamics. Ecological interactions, including the impact of environmental changes on mosquito vectors, reservoirs, predators, and competitors, are critical for understanding the dynamics of broader transmission networks [100]. The adaptive strategies of *Anopheles* mosquitoes in response to environmental changes must also be explored to develop innovative research techniques and interventions to enhance malaria control efforts. As differences in local environments affect mosquito populations and their impact on malaria transmission, control strategies must be tailored to specific regions. By integrating ecological, entomological, and epidemiological data within a One Health framework, researchers can improve predictions of malaria spread and develop region-specific public health strategies. Mathematical modelling further strengthens this approach by forecasting the emergence of new hotspot areas under changing climate scenarios, allowing for timely and proactive interventions.

Finally, future research should address the heterogeneity of malaria infection in macaque populations and critical gaps in understanding mosquito-driven sylvatic transmission cycles. Investigating how variations in macaque infection rates influence transmission dynamics, including potential spillover to human populations, is essential. Additionally, elucidating the ecological and behavioural factors driving sylvatic transmission cycles, particularly the role of specific mosquito species in maintaining and bridging these cycles, will provide critical insights into these complex transmission networks. Climate-responsive surveillance systems are also vital for monitoring vector distribution and enabling timely, context-specific interventions. Most importantly, future research should be directed toward various control methods that can be used to control the larval and adult mosquito stages. Besides, it is also important to study various methods of controlling the parasites in non-human primates.

## 6 Conclusion

In conclusion, zoonotic malaria, particularly the transmission of *P. knowlesi*, is widely recognized as a climate-sensitive disease. However, the evidence suggests that land-use changes play a more significant role than climatic factors like temperature, rainfall, altitude, or seasonality in influencing the behaviour and ecology of *Anopheles* mosquito vectors. Activities such as deforestation, agricultural expansion, and urbanization have been shown to create favourable breeding conditions, alter mosquito habitats, and increase interactions between vectors, human populations, and macaque hosts. These anthropogenic modifications disrupt ecological balances and drive the proliferation and redistribution of malaria vectors, thereby amplifying NHP malaria risks. Moreover, land-use changes can lead to localized microclimate alterations, such as increased temperature, humidity shifts, and changes in water availability, which further influence mosquito survival, development, and biting behaviour. These microclimate shifts may enhance vector suitability in previously unfavourable areas, exacerbating malaria transmission dynamics. It is crucial to recognize land-use change as the primary environmental driver of vector dynamics to design targeted interventions aimed at mitigating the growing threat of NHP malaria.

## CRediT authorship contribution statement

**Rezki Sabrina Masse:** Writing – original draft, Resources, Methodology, Investigation, Funding acquisition, Formal analysis, Data curation. **Indra Vythilingam:** Writing – review & editing, Validation, Supervision, Data curation. **Kimberly Fornace:** Writing – review & editing, Validation, Supervision. **Hidayatulfathi Othman:** Writing – review & editing, Supervision. **Xiaoyue Liu:** Writing – review & editing, Visualization. **Abdul Jabir Jaafar:** Writing – review & editing, Data curation. **Mohd Khadri Shahar:** Writing – review & editing. **Nurul Asmaa Abdul Rahman:** Writing – review & editing, Data curation. **Ainul Huda Khairul Azman:** Writing – review & editing, Data curation. **Nantha Kumar Jeyaprakasam:** Writing – review & editing, Validation, Supervision, Resources, Project administration, Investigation, Funding acquisition, Data curation, Conceptualization.

## Declaration of competing interest

The authors declare the following financial interests/personal relationships which may be considered as potential competing interests:

Nantha Kumar Jeyaprakasam reports article publishing charges was provided by Institute for Medical Research, Malaysia. Rezki Sabrina Masse reports a relationship with Institute for Medical Research, Malaysia that includes: employment. If there are other authors, they declare that they have no known competing financial interests or personal relationships that could have appeared to influence the work reported in this paper.

## Data Availability

All the data used in this review paper are previously published data which are available online
